# Effect of increasing dietary isoacid levels on total tract and apparent ileal nutrient digestibility and fermentation products in growing pigs fed corn-soybean meal diets

**DOI:** 10.1186/s40104-025-01239-0

**Published:** 2025-07-18

**Authors:** Angie P. Benavides-Infante, Lucas A. Rodrigues, Mike T. Socha, Wesley P. Schweer, Crystal L. Levesque, Jorge Y. Perez-Palencia

**Affiliations:** 1 Department of Animal Science, South Dakota State University, 1097 North Campus Drive, Brookings, SD 57007 USA; 2 Zinpro Corporation, Eden Prairie, MN 55344 USA

**Keywords:** Branched-chain fatty acids, Dietary fiber, Feed additives, Isobutyrate, Isovalerate, Volatile fatty acid

## Abstract

**Background:**

Isoacids are the product of branched-chain amino acid fermentation and are naturally produced in the hindgut by microbial fermentation. In ruminants, supplementation of isoacids as a feed additive improves fiber utilization, with a positive impact on animal productivity. However, information on how isoacids impact nutrient digestibility in swine is limited. The objective of this experiment was to determine the optimal inclusion level of an isoacid blend product based on apparent ileal digestibility (AID) and apparent total tract digestibility (ATTD) of nutrients and fermentation products in the ileal digesta and feces of growing pigs fed a corn-soybean meal diet.

**Methods:**

Twelve ileal cannulated pigs (20.9 ± 0.6 kg) were used in a 5-period crossover design with 6 diets and 2 replicate pigs in each period. Dietary treatments consisted of increasing levels (0%, 0.5%, 0.75%, 1%, 1.25%, and 1.5%) of an isoacid blend (isobutyrate, isovalerate, and 2-methyl butyrate, 1:1:1) added to a corn-soybean meal basal diet. Each experimental period consisted of 14 d: 10 d for acclimatization to the diets, 2 d for fecal collection, and 2 d for digesta collection.

**Results:**

Isoacids supplementation quadratically improved the AID of hemicellulose (*P* < 0.05) and tended to improve the AID of crude fiber (*P* < 0.1). The AID of most indispensable amino acids (except Met + Cys, Trp, and Val) as well as the ATTD of crude protein, gross energy, neutral detergent fiber, and hemicellulose improved or tended to improve linear or quadratically (*P* < 0.05 or *P* < 0.1). In addition, the ATTD values of Arg, His, Ile, Leu, Met + Cys, Phe, Thr, and Val increased quadratically (*P* < 0.05). Collectively, 1% of isoacids inclusion resulted in the greatest response. Furthermore, isoacids supplementation tended to increase (*P* = 0.071, quadratically) the concentration of ammonia and decreased (*P* < 0.05) the concentrations of acetic acid and total volatile fatty acids with a tendency to decrease (*P* = 0.064) isovaleric acid in the ileal digesta. In fecal samples, the proportion of acetic acid decreased (*P* < 0.05) quadratically, whereas the proportions of propionic, valeric, and caproic acids tended to increase linearly and/or quadratically (*P* < 0.1).

**Conclusions:**

Isoacids supplementation at 1% in swine diets can improve nutrient digestibility, particularly amino acids and fiber at the ileal level and ATTD of crude protein, gross energy, and hemicellulose.

**Supplementary Information:**

The online version contains supplementary material available at 10.1186/s40104-025-01239-0.

## Background

The swine industry is constantly evolving by implementing new practices and technologies to improve overall productivity and meet sustainability goals [1]. This includes using feed additives and diversifying the ingredients used in the diets to improve efficiencies, reduce feed costs, and consequently promote producer profit [2]. Thus, different feed additives have been developed to improve nutrient utilization, growth performance, reproduction, and health in pigs.

The gastrointestinal tract has a variety of physiological and biochemical functions that contribute to ensuring proper digestion and absorption of nutrients, energy production, immune function, and barrier protection against antigens and pathogens [3]. As key players in gut function, microorganisms produce metabolites, including short-chain fatty acids (SCFA), secondary bile acids, and byproducts of amino acids (AA) metabolism (branched-chain volatile fatty acids, polyamines, indolic compounds, and phenolic compounds), neurotransmitters, and vitamins [4]. The levels of these metabolites are related to important metabolic functions associated with intestinal health, the immune response, and nutrient metabolism, which consequently could influence the growth performance of pigs [4–6]. Short-chain fatty acids (acetate, butyrate, and propionate) are essential for maintaining intestinal health, for example butyrate is the main energy source for colonocytes [7]. Branched-chain volatile fatty acids or isoacids (isobutyrate, isovalerate, and 2-methyl butyrate) are end products of deamination and decarboxylation of branched-chain amino acids (BCAA: Val, Leu, and Ile) by microbial fermentation. Branched-chain fatty acids are present in several organisms as part of more complex lipids and are generated from the undigested protein that reaches the colon. In addition to being used as an energy source by colonocytes [4], isoacids are related to nutrient metabolism and the modulation of immune responses, particularly by attenuating the proinflammatory response [8–10].

An exogenous source of isoacids has been developed through fermentation and is available for supplementation in livestock diets to improve feed utilization, intestinal health, and performance [11]. Dietary inclusion of these isoacids has been investigated, mainly in ruminants, with a focus on rumen fermentation, fiber degradation, and protein synthesis to improve productivity (i.e., milk production) [12, 13]. Depending on the level of inclusion, supplementing dairy cow diets with isoacids improved milk fat content, milk yield, fiber degradability (including neutral detergent fiber digestibility), and the production of volatile fatty acids (VFA) such as acetate [14]. The microorganisms in the rumen synthesize isoacids through oxidative deamination and oxidative decarboxylation of the BCAA. These isoacids are key growth factors for cellulolytic and non-cellulolytic bacteria in the rumen, stimulating cellulose digestion and protein biosynthesis. In turn, cellulolytic bacteria employ an energy source generated from cellulose degradation [15].

Although there is solid evidence of the positive effects of isoacids supplementation in ruminants, studies in swine are limited, and optimal supplementation levels have not been established. We hypothesize that, similar to that in ruminants, dietary supplementation of isoacids in growing pig diets would result in improved nutrient utilization. Therefore, this experiment aimed to determine the optimal inclusion level of an isoacid blend product (isobutyrate, isovalerate, and 2-methyl butyrate) based on nutrient digestibility and intestinal fermentation products in growing pigs fed corn-soybean meal diets.

## Methods

The protocol for this project was approved by the Institutional Animal Care and Use Committee at South Dakota State University (No. 2104-020A).

### Animals and housing

Twelve crossbred pigs (offspring of PIC Camborough sows × Compart Duroc), barrows and gilts, with an initial body weight of 20.9 ± 0.6 kg, were surgically modified with a T-cannula at the distal ileum. One week prior to surgery, pigs were moved from group housing to individual pens for adaptation to individual housing. After surgery, pigs were housed individually in crates for postsurgical care and recovery. One week after surgery, the pigs were moved to individual floor pens in a temperature-controlled room for the entire experimental period. All pens contained one dry self-feeder and one nipple waterer to allow ad libitum access to water.

### Experimental design and dietary treatments

Pigs were used in a replicated 6 × 5 Youden square design with 6 diets, 5 periods, and 2 replicate pigs per diet in each period, which provided 10 observations for each dietary treatment. The experimental diets (Table 1) consisted of graded levels (*n* = 6) of an isoacid blend (isobutyrate, isovalerate, and 2-methyl butyrate – 1:1:1) supplemented to a corn-soybean meal basal diet at 0%, 0.5%, 0.75%, 1%, 1.25%, and 1.5%. Diets were fortified with vitamins and minerals to meet or exceed the nutrient requirements of pigs [16]. Titanium oxide (0.3%) was included in the diets as an indigestible marker to determine nutrient digestibility [17].

**Table 1:** Experimental diet formulation and calculated composition (as-fed basis)^a^

Ingredient, %	Inclusion
Corn	73.83
Soybean meal (46.5%)	23.00
L-Lysine HCl	0.20
L-Threonine	0.06
Monocalcium phosphate	0.90
Limestone	1.18
Salt	0.20
Swine vitamin premix^b^	0.05
Swine mineral premix^c^	0.15
Swine larvicide	0.13
Titanium oxide	0.30
Calculated composition	
ME, kcal/kg	3,350
Crude protein, %	21.0
Crude fiber, %	2.50
SID Lys, %	1.23
SID Arg, %	0.56
SID Met, %	0.36
SID Met + Cys, %	0.68
SID Thr, %	0.73
SID Trp, %	0.20
SID Ile, %	0.63
SID Leu, %	1.23
SID Val, %	0.78
SID His, %	0.42
SID Phe, %	0.72
Calcium, %	0.70
Phosphorus, %	0.60
ATTD P, %	0.29
STTD P, %	0.33
Sodium, %	0.28
Ca/P ratio	1.17

^a^ An isoacid blend (isobutyrate, isovalerate, and 2-methyl butyrate – 1:1:1) was supplemented and mixed on top at 0.5%, 0.75%, 1%, 1.25%, and 1.5% to create five additional dietary treatments. *ATTD* Apparent total tract digestibility, *Ca* Calcium, *ME* Metabolizable energy, *P *Phosphorus, *SID* standard ileal digestibility, *STTD* standardized total tract digestibility

^b^ J & R Distributing Inc., 518 Main Ave, Lake Norden, SD 57248, USA. Minimum provided per kg of diet: Calcium 55 mg, Vitamin A 11,000 IU, Vitamin D_3_ 1,650 IU, Vitamin E 55 IU; Vitamin B_12_ 0.044 mg, Menadione 4.4 mg, Biotin 0.165 mg, Folic Acid 1.1 mg, Niacin 55 mg, d-Pantothenic Acid 60.5 mg, Vitamin B_16_ 3.3 mg, Riboflavin 9.9 mg, Thiamine 3.3 mg

^c^ J & R Distributing Inc., 518 Main Ave, Lake Norden, SD 57248, USA. Minimum provided per kg of diet: Copper 16.5 mg, Manganese 44.1 mg, Selenium 0.03 mg, Zinc 165 mg

### Experimental procedure

The daily animal care procedures included monitoring pig behavior, recording daily room temperature (high and low temperature), feeding, checking waterers and feeders, and cleaning the cannula area. Zinc oxide was used on the skin area around the cannula to minimize irritation from any cannula leakage. Feeding levels were set at 4% of body weight adjusted for individual pig weight at the beginning of each period. Feed was provided each day in 2 equal meals (08:00 and 16:00 h). Each experimental period consisted of 14 d with 10 d for adaptation to the diet, 2 d for feces collection, and the following 2 d for ileal digesta collection. Ileal digesta were collected from 08:00 to 20:00 h into plastic bags with a capacity of 500 mL containing 5 mL of 10% (v/v) formic acid to minimize bacterial fermentation. Every other bag of digesta was collected without formic acid for SCFA analyses. Bags were removed when approximately 70% filled with digesta (or at most every hour) and immediately frozen at −20 °C. At the end of each collection period, the collected feces and digesta were pooled via pig observation, homogenized, subsampled, and stored at −20 °C.

### Chemical analysis

Digesta samples were freeze-dried, and fecal samples were dried in an oven at 102 °C. The digesta and dried fecal samples were ground to pass through a 0.5-mm screen using a mill grinder (Retsch zm 200, ring sieve size: 0.75 mm) before chemical analysis. Dry matter (DM) concentration was determined by drying samples at 102 °C for 24 h in a drying oven [18], and the organic matter (OM) was calculated as the difference between DM and ash. The crude fat (Ether Extraction, AOAC Official Method 920.39; Acid Hydrolysis, AOAC Official Method 954.02, 2006), crude fiber (AOAC Official Method 978.10, 2006), and ash (method 923.03) were also analyzed. Neutral detergent fiber (NDF) was analyzed using the Van Soest fiber fractionation system (JAOAC 56, 1352–1356, 1973). Acid detergent fiber (ADF) and lignin (Acid detergent lignin, ADL) were determined followed AOAC Official Method 973.18 (acid detergent and H_2_SO_4_, respectively). Hemicellulose was calculated as the difference between NDF and ADF, and cellulose was calculated as the difference between ADF and ADL. To determine crude protein (CP) and AA concentrations (including BCAA), the samples were analyzed at a commercial laboratory (University of Missouri, Columbia MO, USA) via method AOAC 990.03 for CP and AOAC Official Method 982.30 E(a,b,c), chp. 45.3.05, 2006 for AA profile. For Met and Cys, were analyzed using the AOAC Official Method 982.30 E(b), chp. 45.3.05, 2006 (performic acid oxidation/acid hydrolysis). The Trp analyze was performed by Alkaline hydrolysis—AOAC Official Method 988.15, chp. 45.4.04, 2006. The gross energy (GE) of the samples was measured using a bomb calorimeter (Parr 6300 calorimeter, Parr Instruments Co., Moline, IL, USA) according to the methods reported by Bech et al. [19]. Briefly, 1 g of ground sample was pressed into a pellet using a pellet press and placed into the bomb calorimeter. Each sample was analyzed in duplicate, and the process was repeated if the difference between the two values was greater than 5%. Titanium concentration was determined by spectrophotometry (model SpectraMAX190, Molecular Devices, Sunnyvale, CA, USA) at 408 nm after ashing at 525 °C for 10 h and digesting with anhydrous sodium sulfate and sulfuric acid at 120 °C for 24 h [18].

### Short-chain fatty acids

Volatile fatty acids and lactic acids, referred to in combination as SCFA, were analyzed in ileal digesta and fecal samples via gas chromatography. All samples were processed according to Darwin et al. [20]. Briefly, samples were thawed, and 2 ± 0.1 g samples were taken, diluted with 4 mL distilled water, vortexed for 3 min and allowed to rest overnight (4 °C). Then, the samples were centrifuged at 4,000 × *g* for 5 min, and the supernatant was filtered through a 0.22-µm pore size filter (Millex-GP). The upper layer (0.57 mL) was acidified with 0.15 mL of internal standard (5 mmol/L, 4-methyl-valeric acid, 277827, Sigma, St. Louis, MO, USA), acidified with 0.48 mL of periodic acid (100 mmol/L) and 0.3 mL of formic acid (10% w/v), and incubated at 100 °C for 1 h. The mixed solution was collected and used for SCFA determination using a 6890 N Network GC System gas chromatograph (Agilent Technologies) equipped with a flame ionization detector, according to Izuddin et al. [21]. One microliter of the sample was injected at a split ratio of 1:30 at a temperature of 230 °C. Separation of VFA profile was determined using Quadrex 007–10 Series (Quadrex Corp., New Haven, CT 06525, USA) bonded-phase fused silica capillary column (15 m, 0.250 mm internal diameter, 0.25 μm film thickness). The temperature of the column was set at 60 °C for 2 min, increased to 100 °C (10 °C/min), increased to 200 °C (20 °C/min), and held for 2 min. Nitrogen gas was supplied as carrier gas at a rate of 1 mL/min. The temperature of the detector was set at 230 °C. Commercial standards (Sigma-Aldrich, St. Louis, MO, USA) of lactic (1356734), acetic (45997), propionic (94425), isobutyric (46935), butyric (19215), isovaleric (78651), valeric (75054), and caproic (21529) acids were used as external standards for gravimetrically prepare a calibration curve and peak identification. The molar concentration of SCFA was identified on the basis of a single point of the internal standard and a calibration curve with external standards.

### Ammonia

Ammonia was analyzed in the ileal digesta and fecal samples using the method described by Novamsky et al. [22]. This method involves the conversion of both ammonia and organic nitrogen-based compounds to ammonia. These compounds were combined with hypochlorite to form monochloramine. Furthermore, the monochloramine reacts with salicylate and nitropyridine to produce a blue-colored compound called indosalicylate. The intensity of the resulting color was proportional to the concentration of ammonia. The samples were prepared by mixing them with water. Before analysis, the water portion of each sample was separated via centrifugation. A 1-mL aliquot of the supernatant was used for the analysis; this mixture was diluted with 9 mL of deionized water, and then 5.0 mL of the sample was transferred to a TNTplus Vial Test, HR (2–47 mg/L NH₃-N). The analysis was conducted for ammonia-N using the DR3900 spectrophotometer, and the results were multiplied by the dilution factor.

### Calculations and statistical analysis

Apparent ileal digestibility (AID) values were calculated from the difference between the dietary intake of the nutrient and the concentration of the nutrient in the digesta present in the distal ileum of pigs according to the following equation [23, 24]:

$$\mathrm{AID},\;\%\;=100-\left[100\times\left(\frac{{\mathrm{Ti}}_{\mathrm d}\times{\mathrm N}_{\mathrm i}}{{\mathrm{Ti}}_{\mathrm i}\times{\mathrm N}_{\mathrm d}}\right)\right]$$

where Ti_d_ = the concentration of titanium in the diet; Ti_i_ = the concentration of titanium in the ileal sample; N_i_ = the concentration of the nutrient in the ileal sample; and N_d_ = the concentration of the nutrient in the diet.

To determine the apparent total tract digestibility (ATTD) of nutrients, the following equation described by Adeola [25] was used:

$$\mathrm{ATTD},\;\%\;=\;100-\left[\frac{\left({\mathrm{Ti}}_{\mathrm d}\times{\mathrm N}_{\mathrm f}\right)}{{\mathrm{Ti}}_{\mathrm f}\times{\mathrm N}_{\mathrm d}}\right]\times100$$

where digestibility refers to ATTD; Ti_d_ and Ti_f_ represent the concentrations of marker compounds in the diet and feces, respectively; and N_d_ and N_f_ represent the concentrations of nutrients in the feed and feces, respectively.

The UNIVARIATE procedure of SAS (Version 9.4; SAS Inst., Inc., Cary, NC, USA) was used to confirm the homogeneity of variance and to analyze outliers. The data were analyzed using MIXED procedure of SAS, with dietary treatment as the main effect, period as a random effect and pigs as the experimental unit. Orthogonal polynomial contrasts were used to determine the linear, quadratic, and cubic effects of increasing levels of isoacids. In addition, preplanned contrasts were used to compare 0% inclusion with isoacid-supplemented groups. For all the statistical tests, differences were considered significant at *P* ≤ 0.05 and tendency at 0.05 < *P* ≤ 0.10.

## Results

The analyzed composition of the experimental diets used in this experiment are presented in Table 2. Overall, the analyzed chemical composition reflected the targets in the formulation. In particular, the nutrient profile across the experimental diets was not different.

**Table 2:** Analyzed composition of experimental diets (as-fed basis)^a^

Item	Isoacids inclusion					
	**0%**	**0.5%**	**0.75%**	**1%**	**1.25%**	**1.5%**
Crude protein, %	17.36	16.06	19.90	19.41	17.18	16.40
Dry matter, %	89.22	88.51	89.08	89.26	88.42	88.83
Gross energy, kcal/kg	3,786	3,759	3,822	3,872.5	3,762	3,772
Crude fat, %	2.38	1.45	1.52	1.46	1.29	1.25
Crude fiber, %	2.13	1.85	1.96	2.67	2.63	1.97
Ash, %	4.48	5.00	5.22	5.39	5.21	5.44
NDF, %	8.04	7.65	7.63	8.10	7.62	6.95
ADF, %	3.54	3.28	3.29	3.39	3.09	3.00
Cellulose, %	3.32	3.07	3.11	3.26	2.97	2.85
ADL, %	0.22	0.21	0.18	0.13	0.12	0.15
Hemicellulose, %	4.50	4.37	4.34	4.71	4.53	3.95
Total AA, %						
Asp	1.75	1.64	1.91	1.98	1.64	1.57
Thr	0.68	0.66	0.77	0.81	0.69	0.64
Ser	0.75	0.70	0.80	0.83	0.72	0.66
Glu	3.36	3.18	3.59	3.69	3.19	3.05
Pro	1.04	1.00	1.08	1.11	1.00	0.96
Gly	0.74	0.69	0.78	0.80	0.68	0.67
Ala	0.90	0.86	0.94	0.96	0.87	0.83
Cys	0.31	0.30	0.31	0.33	0.29	0.28
Val	0.88	0.83	0.92	0.94	0.81	0.80
Met	0.28	0.26	0.28	0.29	0.25	0.25
Ile	0.80	0.75	0.85	0.88	0.74	0.72
Leu	1.53	1.48	1.62	1.66	1.49	1.42
Tyr	0.58	0.56	0.62	0.65	0.56	0.54
Phe	0.88	0.84	0.94	0.97	0.83	0.80
Lys	1.16	1.09	1.18	1.25	1.06	1.03
His	0.48	0.45	0.50	0.52	0.44	0.44
Arg	1.15	1.08	1.22	1.26	1.05	1.03
Trp	0.20	0.21	0.22	0.22	0.19	0.18

^a^An isoacid blend (isobutyrate, isovalerate, and 2-methyl butyrate – 1:1:1) was supplemented at 0.5%, 0.75%, 1%, 1.25%, and 1.5% to create five additional dietary treatments. *ADF* Acid detergent fiber, *ADL* Acid detergent lignin, *NDF* Neutral detergent fiber

Body weight data is presented as a supplemental file (Additional file 1) and indicates no differences across dietary treatments.

### Nutrient digestibility

The effects of increasing levels of isoacids supplementation on AID and ATTD are shown in Tables 3 and 4, respectively. The AID of crude fiber (*P* = 0.080) and hemicellulose (*P* < 0.05) increased quadratically, where 1% isoacid inclusion promoted the greatest response. Dietary supplementation with isoacids improved (*P* < 0.05, linear and/or quadratic) AID of Ile, Phe, Thr and Tyr with tendencies to improve AID of Arg, His, Lys, Met, and the mean for indispensable AA as well as some dispensable AAs (Ala, Glu, and Gly). The ATTD of CP, GE, and hemicellulose increased linearly and/or quadratically (*P* < 0.05) in response to isoacids supplementation while the ATTD of NDF tended to decrease (*P* = 0.063). The ATTD of Arg, His, Ile, Leu, Met + Cys, Phe, Thr, Val, and most of the dispensable AA (except Ala) increased quadratically (*P* < 0.05), where the greatest response was observed between 0.75% and 1% isoacids in the diet. Considering the contrast analysis (0% inclusion vs. isoacids supplementation), the dietary inclusion of isoacids improved the AID of ash, hemicellulose, Ile, Leu, Me, Phe, Thr and Tyr (*P* < 0.05) as well as the ATTD of hemicellulose and the majority of indispensable and dispensable AA.

**Table 3:** Apparent ileal digestibility (%) of nutrients and amino acids at different levels of isoacids inclusion in the diet of growing pigs^a^

Item	**Isoacids inclusion, %**						**SEM**	***P*-value**		
	**0**	**0.5**	**0.75**	**1**	**1.25**	**1.5**		**Linear**	**Quadratic**	**0% vs. IsoSup**^**b**^
CP	79.65	80.68	82.25	81.81	79.69	80.46	0.691	0.835	0.459	0.763
DM	77.90	78.72	78.67	79.40	77.42	78.79	0.531	0.412	0.183	0.806
OM	79.39	80.88	80.31	81.05	79.24	80.21	0.492	0.536	0.473	0.788
GE	80.58	81.48	81.49	82.09	80.07	81.67	0.476	0.818	0.819	0.903
Crude fat	54.99	50.28	50.08	47.68	51.18	56.35	4.367	0.025	0.658	0.024
Crude fiber	53.35	50.54	54.19	61.48	55.80	53.54	2.63	0.560	0.080	0.224
Ash	55.42	60.81	58.62	64.10	54.64	63.24	1.118	0.056	0.108	0.025
NDF	49.63	50.32	45.48	52.30	44.82	46.27	1.758	0.529	0.474	0.906
ADF	48.15	43.63	46.75	47.63	43.95	46.02	1.872	0.234	0.893	0.582
Cellulose	34.84	34.07	36.06	35.92	35.13	36.45	1.844	0.196	0.408	0.359
ADL	44.63	48.50	38.41	50.60	40.01	40.90	1.792	0.533	0.128	0.675
Hemicellulose	44.83	46.04	48.12	50.36	47.08	45.70	3.349	0.189	0.039	0.041
Indispensable AA										
Arg	81.05	80.92	82.78	85.00	82.13	81.39	1.065	0.358	0.099	0.260
His	76.18	76.34	78.15	80.66	77.28	78.52	1.163	0.084	0.357	0.139
Ile	68.91	71.30	72.83	75.26	72.33	72.59	1.412	0.035	0.081	0.019
Leu	71.44	73.64	74.76	76.75	75.62	75.42	1.276	0.010	0.187	0.013
Lys	68.65	70.69	70.83	75.10	71.97	71.62	1.550	0.078	0.196	0.077
Met	75.89	78.58	78.24	80.20	78.66	79.18	1.273	0.067	0.249	0.041
Met + Cys	67.98	70.12	67.90	72.25	70.10	70.70	1.441	0.153	0.703	0.207
Phe	70.69	72.64	73.90	76.17	74.03	73.92	1.303	0.033	0.124	0.026
Thr	56.29	58.90	61.09	66.29	60.90	60.35	1.551	0.016	0.017	0.008
Trp	71.66	72.33	71.52	74.52	72.17	72.16	1.452	0.664	0.590	0.619
Val	66.65	68.86	68.69	71.49	68.21	69.39	1.434	0.203	0.263	0.131
Mean	70.89	72.26	73.28	76.15	73.22	73.46	1.230	0.063	0.155	0.055
Dispensable AA										
Ala	61.69	64.12	64.96	67.71	65.79	65.41	1.798	0.081	0.252	0.079
Asp	68.72	68.74	70.28	73.54	70.22	70.18	1.362	0.201	0.304	0.262
Cys	59.07	60.30	57.56	64.29	61.54	62.22	1.842	0.122	0.891	0.348
Glu	75.91	78.37	77.57	79.54	79.53	79.18	1.414	0.064	0.518	0.078
Gly	45.14	46.19	48.91	58.40	48.84	46.43	3.032	0.304	0.077	0.194
Ser	66.17	65.97	69.30	71.52	68.37	67.71	1.474	0.155	0.147	0.162
Tyr	70.62	72.74	73.86	76.37	74.52	74.25	1.290	0.015	0.132	0.015
Mean	62.22	64.48	64.37	67.63	66.37	65.01	1.753	0.109	0.287	0.103
Total AA mean	67.76	69.24	70.32	73.55	69.66	69.84	1.727	0.263	0.186	0.171

^a^ An isoacids blend was included at 0.5%, 0.75%, 1%, 1.25%, and 1.50% in a corn-soybean meal basal diet. *AA* Amino acids, *ADF* Acid detergent fiber, *ADL* Acid detergent lignin, *CP* Crude protein, *DM* Dry matter, *GE* Gross energy, *OM* Organic matter, *NDF *Neutral detergent fiber

^b^ This contrast specifically compared the basal diet (0% inclusion) vs. isoacids-supplemented diets (0.5%, 0.75%, 1%, 1.25%, and 1.5% inclusion)

**Table 4:** Apparent total tract digestibility (%) of nutrients and amino acids at different levels of isoacids inclusion in the diet of growing pigs^a^

Item	Isoacids inclusion, %						SEM	*P*-value		
	**0**	**0.5**	**0.75**	**1.0**	**1.25**	**1.5**		**Linear**	**Quadratic**	**0% vs. IsoSup**^**b**^
CP	87.38	88.37	89.99	89.21	87.05	88.03	0.747	0.952	0.029	0.186
DM	87.82	88.45	88.20	88.42	86.50	88.33	0.593	0.634	0.635	0.818
OM	89.64	91.01	90.16	90.05	88.66	90.05	0.616	0.464	0.352	0.626
GE	87.28	88.11	88.03	88.39	86.36	88.22	0.601	0.013	0.013	0.438
Crude fat	57.54	52.57	52.33	49.74	53.44	58.88	4.141	0.997	0.120	0.393
Crude fiber	66.69	59.5	63.65	71.70	65.31	62.89	2.153	0.890	0.988	0.405
Ash	57.10	62.63	60.35	65.92	56.22	65.11	2.082	0.100	0.413	0.044
NDF	62.59	63.22	56.99	65.05	55.95	57.98	2.192	0.063	0.629	0.280
ADF	72.84	65.12	69.16	68.63	64.16	68.08	2.477	0.140	0.259	0.047
Cellulose	51.55	49.13	49.74	48.35	50.88	50.21	1.089	0.141	0.118	0.169
ADL	51.46	55.78	44.10	57.79	45.82	46.96	2.926	0.165	0.364	0.685
Hemicellulose	58.14	59.42	60.78	62.65	61.22	59.33	1.915	0.871	0.022	0.030
Indispensable AA										
Arg	82.30	83.16	84.74	84.45	80.11	81.68	1.058	0.274	0.031	0.663
His	77.34	78.66	80.46	80.52	75.59	77.74	1.059	0.632	0.019	0.308
Ile	50.30	56.58	58.60	58.61	52.31	56.79	1.999	0.142	0.023	0.009
Leu	60.53	66.13	66.36	65.55	62.98	65.96	1.464	0.096	0.050	0.006
Lys	55.34	60.64	60.38	61.73	56.74	60.23	2.133	0.316	0.135	0.066
Met	47.11	51.55	54.11	53.25	48.37	52.95	2.165	0.224	0.145	0.054
Met + Cys	55.34	60.19	61.28	61.49	56.73	59.94	1.620	0.218	0.033	0.018
Phe	59.66	64.78	66.32	66.37	60.64	63.20	1.576	0.410	0.004	0.014
Thr	50.16	55.50	59.44	60.96	53.75	56.46	1.669	0.027	0.002	0.001
Trp	79.08	83.10	82.37	81.72	80.18	82.18	1.079	0.326	0.093	0.027
Val	47.87	54.81	56.09	55.49	49.42	54.87	2.028	0.167	0.036	0.010
Mean	61.06	65.49	66.89	66.87	62.07	65.64	1.434	0.171	0.024	0.012
Dispensable AA										
Ala	30.78	41.42	40.08	38.70	37.66	41.05	3.080	0.114	0.186	0.030
Asp	63.25	67.01	70.08	70.15	64.20	66.17	1.592	0.409	0.005	0.024
Cys	63.49	68.84	68.45	69.73	64.23	65.76	1.400	0.701	0.002	0.019
Glu	72.52	76.95	77.61	77.66	74.29	76.11	1.161	0.160	0.007	0.004
Gly	43.14	51.40	52.94	52.78	46.26	51.88	2.374	0.074	0.025	0.004
Ser	68.14	69.67	72.06	72.53	67.23	68.47	1.289	0.872	0.012	0.219
Tyr	58.17	65.44	66.91	68.36	64.68	66.76	1.905	0.005	0.016	0.001
Mean	62.82	67.39	67.90	68.24	64.21	66.74	1.322	0.196	0.016	0.010
Total AA mean	61.28	66.63	67.78	67.90	63.31	66.29	1.484	0.120	0.012	0.004

^a ^An isoacid blend was included at 0.5%, 0.75%, 1%, 1.25%, and 1.50% in a corn-soybean meal basal diet. *AA* Amino acids, *ADF* Acid detergent fiber, *ADL* Acid detergent lignin, *CP* Crude protein, *DM* Dry matter, *GE* Gross energy, *OM* Organic matter, *NDF* Neutral detergent fiber

^b^ This contrast specifically compared the basal diet (0% inclusion) vs. isoacids-supplemented diets (0.5%, 0.75%, 1%, 1.25%, and 1.5% inclusion)

### Fermentation products

Inclusion of isoacids quadratically tended to increase (*P* = 0.071) the concentration of ammonia and decreased (*P* < 0.05) the concentrations of acetic acid and total VFA with a tendency to also decrease (*P* = 0.064) isovaleric acid in the ileal digesta samples (Table 5). In addition, as a proportion of the total VFA content, the acetic acid (*P* < 0.05) and isovaleric acid (*P* = 0.09) contents decreased, whereas the propionic acid content increased quadratically (*P* < 0.05). The dietary inclusion of isoacids did not have a major effect on the fecal VFA concentration (Table 6). However, the proportion of acetic acid decreased (*P* < 0.05) quadratically, whereas the proportions of propionic, valeric, and caproic acids tended to increase quadratically (*P* < 0.1). Considering the contrast analysis (0% inclusion vs. isoacid supplementation), isoacids supplementation increased (*P* < 0.05) the concentrations of ammonia in ileal digesta samples and caproic acid in fecal samples, increased the proportions of propionic and caproic acids (*P* < 0.05) and decreased the proportion of acetic acid (*P* = 0.070).

**Table 5:** Concentrations of ammonia and volatile fatty acids (VFA) in the ileal digesta samples at different levels of isoacids inclusion in the diet of growing pigs^a^

Item	Isoacids inclusion, %						SEM	*P*-value		
	**0**	**0.5**	**0.75**	**1**	**1.25**	**1.5**		**Linear**	**Quadratic**	**0% vs. IsoSup**^**b**^
Ammonia	172.47	235.80	229.10	269.90	196.70	226.20	25.609	0.259	0.071	0.050
VFA, mmol/L										
Lactic	1.68	1.27	1.49	1.31	1.36	0.76	0.388	0.182	0.686	0.326
Acetic C2	20.07	15.18	13.82	15.93	16.92	22.07	2.048	0.564	0.002	0.170
Propionic C3	6.84	6.18	5.87	6.64	6.10	6.92	0.642	0.987	0.262	0.505
Isobutyric iC4	0.15	0.19	0.10	0.15	0.31	0.20	0.108	0.525	0.741	0.769
Butyric C4	2.58	2.42	2.01	2.20	2.32	2.50	0.300	0.755	0.220	0.415
Isovaleric C5	0.33	0.02	0.09	0.18	0.18	0.26	0.111	0.948	0.064	0.162
Valeric C5	0.11	0.22	0.11	0.12	0.11	0.22	0.127	0.809	0.885	0.750
Caproic C6	0.01	0.15	0.13	0.00	0.00	0.13	0.090	0.874	0.732	0.479
Total VFA	30.09	24.31	22.12	25.37	25.94	32.30	2.569	0.632	0.003	0.174
VFA, % of total										
Acetic C2	64.58	62.41	61.92	61.88	64.83	67.54	2.041	0.315	0.040	0.713
Propionic C3	24.14	26.02	26.92	26.98	23.90	22.10	1.879	0.433	0.048	0.631
Isobutyric iC4	0.71	0.47	0.39	0.38	1.04	0.59	0.402	0.832	0.523	0.761
Butyric C4	8.92	9.18	9.28	8.91	8.99	7.80	0.752	0.376	0.273	0.920
Isovaleric C5	1.35	0.19	0.36	0.67	0.69	0.86	0.438	0.646	0.090	0.117
Valeric C5	0.23	1.11	0.51	0.64	0.55	0.75	0.570	0.738	0.658	0.462
Caproic C6	0.02	0.63	0.63	0.00	0.00	0.37	0.344	0.927	0.449	0.453

^a^ An isoacid blend was included at 0.5%, 0.75%, 1%, 1.25%, and 1.50% in a corn-soybean meal basal diet

^b^ This contrast specifically compared the basal diet (0% inclusion) vs. isoacids-supplemented diets (0.5%, 0.75%, 1%, 1.25%, and 1.5% inclusion)

**Table 6:** Concentration of ammonia and volatile fatty acids (VFA) in fecal samples at different levels of isoacids inclusion in the diet of growing pigs^a^

Item	Isoacids inclusion, %						SEM	*P*-value		
	**0**	**0.5**	**0.75**	**1**	**1.25**	**1.5**		**Linear**	**Quadratic**	**0% vs. IsoSup**^**b**^
Ammonia	206.91	200	202	206.9	200.5	197.4	11.272	0.654	0.946	0.670
VFA, mmol/L										
Lactic	3.61	3.23	3.63	3.62	2.62	3.69	0.366	0.606	0.688	0.552
Acetic C2	30.98	28.82	30.64	25.16	30.76	28.99	2.101	0.540	0.446	0.389
Propionic C3	8.06	8.75	9.02	7.99	8.84	9.15	0.580	0.319	0.962	0.306
Isobutyric iC4	2.27	2.25	2.33	2.22	2.30	2.32	0.100	0.724	0.829	0.869
Butyric C4	4.16	4.29	4.47	4.06	4.30	4.44	0.361	0.724	0.975	0.721
Isovaleric C5	1.94	1.91	1.95	1.88	1.96	1.90	0.088	0.847	0.934	0.809
Valeric C5	1.93	2.06	2.15	2.08	2.16	2.09	0.095	0.173	0.296	0.111
Caproic C6	0.59	1.07	1.06	1.17	0.96	1.09	0.186	0.102	0.138	0.028
Total VFA	49.98	49.15	51.63	44.56	52.50	49.31	2.987	0.967	0.757	0.874
VFA, % of total										
Acetic C2	61.62	58.54	59.09	56.21	58.67	60.15	1.453	0.305	0.042	0.070
Propionic C3	16.33	17.70	17.50	18.00	17.60	16.98	0.467	0.248	0.019	0.028
Isobutyric iC4	4.64	4.62	4.58	5.07	4.57	4.52	0.221	0.917	0.453	0.911
Butyric C4	8.32	8.73	8.66	9.05	9.06	8.37	0.592	0.689	0.416	0.511
Isovaleric C5	4.00	3.94	3.84	4.29	3.91	3.75	0.207	0.657	0.500	0.838
Valeric C5	3.99	4.22	4.20	4.74	4.24	4.06	0.207	0.473	0.082	0.205
Caproic C6	1.09	2.26	2.14	2.65	1.95	2.17	0.401	0.091	0.069	0.017

^a^ An isoacid blend was included at 0.5%, 0.75%, 1%, 1.25%, and 1.50% in a corn-soybean meal basal diet

^b ^This contrast specifically compared the basal diet (0% inclusion) vs. isoacids-supplemented diets (0.5%, 0.75%, 1%, 1.25%, and 1.5% inclusion)

## Discussion

The use of isoacids as a feed additive for swine represents a novel approach to possibly improve nutrient utilization in swine diets, especially when considering the effects on fiber digestion, which may allow for the use of higher inclusion rates of alternative ingredients characterized by high fiber concentration. To the best of our knowledge, this experiment represents the first effort to determine the optimal inclusion levels of isoacids as feed additives for swine based on dietary nutrient digestibility.

Overall, including 1% of an isoacid blend in corn-soybean meal diets fed to growing pigs promoted the greatest responses in terms of nutrient digestibility and fermentation products; however, benefits of isoacids supplementation were observed from 0.5% to 1.5% inclusion. When included in ruminant diets, isoacids are associated with proliferation of fiber-digesting organisms in the rumen and can be used in the biosynthesis of BCAA [12, 26]. As a result, dietary supplementation with isoacids has been shown to improve fiber digestibility [27–29] and feed utilization in ruminants [13, 30]. In pigs, it was expected that isoacids supplementation would benefit microbial activity and function in the hindgut, resulting in improved nutrient digestion, particularly at the total tract level. This is due to the low capacity of pigs to utilize fiber, especially in the early growth phases [31]. This experiment supports that the benefits of isoacids supplementation on nutrient digestibility can also be observed in the small intestine and in relatively low-fiber diets. Like in ruminants, isoacids were associated with improvements in fiber digestibility at the ileal and total tract level, particularly hemicellulose. Consequently, improved digestibility of AA, energy, and other nutrients was observed at both the ileal and total tract levels. Nonetheless, the mechanism of action through which isoacids promoted nutrient and fiber digestion cannot be determined from the present experiment.

At the total tract level, a tendency for a negative effect of isoacids on ATTD of some fiber components, particularly NDF, was observed. Although this response could have been driven by the response values from the two highest inclusion levels of isoacids supplementation, the contrast (0% vs. IsoSup) was not significant, which indicates that isoacids supplementation not necessarily will result in negative effects over fiber digestion, rather could be a maximum level where no additional benefits are observed. Moreover, since isoacids are presumably an energy source for cellulolytic microbes, there may be an inclusion rate beyond which responses decline due to an oversupply of resources exceeding microbial metabolic capacity.

Nondigestible carbohydrates, such as nonstarch polysaccharides (cellulose, pectin, and hemicellulose), resistant starch, and nondigestible oligosaccharides, are substrates for the production of SCFA by microbial fermentation (*Bacteroides* and *Ruminococcus)* in the large intestine [32]. This type of nondigestible carbohydrate is present in the cell wall of cereals and grains (i.e., corn and soybean meal) that are included in swine diets. Thus, it is likely that the inclusion of isoacids as feed additives has the potential to modulate the intestinal microbiota by increasing the population of hemicellulose-degrading bacteria, increasing the digestibility of hemicellulose. In this regard, Wang et al. [33] supplemented isobutyrate in diets for steers at 8.4, 16.8, and 25.2 g per steer per day and reported improved cellulose and hemicellulose degradation. These results were attributed to the change in the microbiome with increased activity of microbial enzymes related to the degradation of these components. In a recently published work, Kerr et al. [34] reported that the addition of isobutyrate to diets for finisher pigs increased the ATTD of GE and nitrogen in diets containing 40% of distillers dried grains with solubles. However, in their study, the addition of isoacids had no effect on the fecal microbial ecology or VFA concentrations.

Isoacids also improved the AID of most indispensable and dispensable AA and increased the ATTD of all AA (except Lys, Met, and Ala). The improvement in the digestibility of AA and other nutrients may be related to the increase in the degradation of fiber components. Owing to the lack of endogenous enzymes capable of hydrolyzing fiber components, the inclusion of fiber in growing pig diets is typically limited and has been associated with negative effects on nutrient digestibility and, in some cases, compromised growth performance [31, 35–37]. Thus, by increasing fiber digestibility, isoacids supplementation may reduce the negative effects of dietary fiber and improve the utilization of other nutrients. For example, Dilger et al. [38] evaluated the effects of increasing levels of soybean hulls (3%, 6%, and 9%) on the nitrogen and AA digestibility of cornstarch-SBM diets fed to growing pigs. The authors reported that the digestibility of several AA (Arg, His, Ile, Lys, Phe, and Trp) decreased linearly, indicating that for every 1% increase in soybean hulls, the digestibility of these AA decreased by 0.2%.

On the other hand, CP and AA analyzed content varied across experimental diets. For example, CP varied from 16.06% to 19.90% with the higher content for the 0.75% and 1% isoacids inclusion. This could be considered as a potential limitation of this study. However, an increase in dietary CP content is not correlated with increased CP or AA digestibility in pigs [39, 40].

In the present experiment, the changes in microbial fermentation were indirectly evaluated via VFA concentrations in the ileal and fecal samples. There were no substantial effects on the VFA concentrations, which may be related to the level and source of dietary fiber provided in the diet, considering that SCFA levels are affected mainly by the type, fermentability, and digestibility of the fiber [41]. However, the results revealed a quadratic response for acetic and propionic acid concentrations where the acetic acid concentration decreased and the propionic acid concentration increased. Short-chain fatty acids, particularly acetate, propionate, and butyrate, are considered important health-related microbial metabolites associated with regulatory functions on intestinal barrier function, immunity, and metabolism [42, 43]. In this context, Wu et al. [44] demonstrated that propionate is a key metabolite responsible for the amelioration of intestinal inflammation and dysbiosis. In humans, propionate can inhibit intestinal inflammation and reduce the expression of proinflammatory cytokines TNF-α and interleukin 6 [45]. Our results can be related to those of Liu et al. [46], who supplemented isobutyrate in the diet for steers at 8.4, 16.8, and 25.2 g per steer per day based on a DM intake. They reported a linear and quadratic increase in the acetate to propionate ratio as isobutyrate supplementation increased, indicating that the optimum daily amount was approximately 16.8 g per steer. These results indicated that isobutyrate supplementation alters the rumen fermentation pattern, favoring bacterial acetate producers. Similarly, Liu et al. [13] reported that the supplementation of isobutyrate to cows at 20 g, 40 g, and 60 g per day per cow based on DM intake generated positive changes in the VFA profile, which was associated with an increase in milk yield at the 40 g inclusion level. In a more recent study, Liu et al. [47] reported improvements in gain, ruminal total VFA concentration, and total tract nutrient digestibility when an isoacid blend (isobutyrate, isovalerate and 2-methylbutyrate at a ratio of 1:1:1) was supplemented in the diet of 10-month-old calves. In addition, Quispe et al. [48] reported increased acetate production when sheep were fed an isoacid blend (isobutyrate, 2-methyl butyrate, valerate, and isovalerate) mixed in rations at 0.07 g and 0.14 g/kg BW per day. Based on the changes in fermentation products observed in this experiment, the modulation of the intestinal microbiota through the gastrointestinal tract could in part explain the effects of isoacids on nutrient digestibility. However, the direct effects of isoacids supplementation on the intestinal microbiota must be better understood.

## Conclusions

Dietary supplementation with isoacids (isobutyrate, isovalerate, and 2-methyl butyrate, 1:1:1) at 1% can improve dietary nutrient digestibility, particularly ileal digestibility of indispensable AA (Ile, Leu, Phe, and Thr) and hemicellulose and total tract digestibility of CP, GE, and hemicellulose. Furthermore, pigs fed isoacids had lower concentration of fermentation products at the ileal (i.e., acetic acid, isovaleric acid) and greater concentrations at the fecal (i.e., propionic, valeric, and caproic acids) level.

## Supplementary Information

Additional file 1. Average body weight of growing pigs fed diets with different levels of isoacids inclusion.

 

## Data Availability

The datasets used and/or analyzed during the current experiment are available from the corresponding author on reasonable request**.**

## Abbreviations:

AA: Amino acids
ADF: Acid detergent fiber
ADL: Acid detergent lignin
AID: Apparent ileal digestibility
ATTD: Apparent total tract digestibility
BCAA: Branched-chain amino acids
CP: Crude protein
DM: Dry matter
GE: Gross energy
ME: Metabolized energy
NDF: Neutral detergent fiber
OM: Organic matter
SCFA: Short-chain fatty acids
SID: Standardized ileal digestible
VFA: Volatile fatty acids
