# Supplementary material for: Effect of increasing dietary isoacid levels on total tract and apparent ileal nutrient digestibility and fermentation products in growing pigs fed corn-soybean meal diets
Source: J Anim Sci Biotechnol. 2025 Jul 18;16:102. doi: 10.1186/s40104-025-01239-0 (PMC12272956; doi:10.1186/s40104-025-01239-0)
Supplement: Supplementary file 1 — Additional file 1. Average body weight of growing pigs fed diets with different levels of isoacids inclusion. [file 40104_2025_1239_MOESM1_ESM.docx]

**Additional file 1** Average body weight of growing pigs fed diets with different levels of isoacids inclusion

| **Body weight** | **Isoacids inclusion, %** | | | | | |
| --- | --- | --- | --- | --- | --- | --- |
|  | **0** | **0.5** | **0.75** | **1** | **1.25** | **1.5** |
| Period 1 | 20.8 | 20.5 | 20.8 | 20.5 | 20.5 | 20.8 |
| Period 2 | 26.3 | 26.0 | 25.8 | 25.8 | 26.5 | 26.5 |
| Period 3 | 33.0 | 32.8 | 33.4 | 32.8 | 32.4 | 31.9 |
| Period 4 | 40.3 | 40.0 | 40.3 | 40.8 | 39.8 | 39.3 |
| Period 5 | 48.5 | 47.8 | 48.6 | 48.9 | 47.8 | 47.4 |
| Overall average | 33.7 | 33.4 | 33.8 | 33.7 | 33.4 | 33.2 |
| Standard deviation | 11.0 | 10.9 | 11.1 | 11.4 | 10.8 | 10.5 |
